# UBE2L3 expression in human gastric cancer and its clinical significance

**DOI:** 10.1007/s00432-024-05669-7

**Published:** 2024-04-24

**Authors:** Xiaoxia Zhang, Yujie Wei, Fanqi Wu, Mei Li, Cong Han, Chengdong Huo, Zhi Li, Futian Tang, Wenting He, Yang Zhao, Yumin Li

**Affiliations:** 1 Department of the Second Hospital & Clinical Medical School, Lanzhou University, Lanzhou, 730030 China; 2 Key Laboratory of Digestive System Tumors of Gansu Province, Lanzhou, 730030 China; 3 Department of Ophthalmology, The Second Hospital & Clinical Medical School, Lanzhou University, Lanzhou, 730030 China; 4 Department of General Surgery, The Second Hospital & Clinical Medical School, Lanzhou University, Lanzhou, 730030 China; 5 Department of Pneumology, The Second Hospital & Clinical Medical School, Lanzhou University, Lanzhou, 730030 China

**Keywords:** UBE2L3, Gastric cancer, Oncogenes, Ubiquitin-conjugating enzyme E2

## Abstract

**Purpose:**

Gastric cancer (GC) is prevalent as one of the most common malignant tumors globally, with a particularly high incidence in China. The role of UBE2L3 in the initiation and progression of various cancers has been well documented, but its specific significance in GC is not yet fully elucidated. The objective of this study is to examine the expression and importance of UBE2L3 in human gastric cancer tissues.

**Methods:**

Immunohistochemical staining and survival analysis were conducted on 125 cases of GC. Western blot and quantitative real-time polymerase chain reaction (qRT-PCR) were employed to assess the expression of UBE2L3 in GC cell lines. Cell lines with UBE2L3 knockdown and overexpression were cultured through lentivirus transfection and subsequently assessed using Western blot analysis. The involvement of UBE2L3 in the proliferation, invasion, and apoptosis of GC cells was confirmed through in vitro experiments, and its capacity to facilitate tumor growth was also validated in in vivo studies.

**Results:**

The up-regulation of UBE2L3 expression was observed in GC, and its high expression was found to be significantly associated with the degree of differentiation (χ^2^ = 6.153, *P* = 0.0131), TNM stage (χ^2^ = 6.216, *P* = 0.0447), and poor overall survival. In vitro, UBE2L3 has been shown to enhance functions in GC cell lines, such as promoting proliferation and invasion, and inhibiting apoptosis. In vivo experiments have validated the role of UBE2L3 in promoting tumor growth.

**Conclusions:**

The findings of our study demonstrate the significant involvement of UBE2L3 in the pathogenesis and advancement of gastric cancer, suggesting its potential as a therapeutic target.

**Supplementary Information:**

The online version contains supplementary material available at 10.1007/s00432-024-05669-7.

## Introduction

Gastric cancer (GC) is a widespread disease and a significant threat to human health (Bray et al. 2018; Smyth et al. 2020). According to the GLOBOCAN 2020 report, the incidence of GC ranked fifth and its mortality ranked fourth among all types of tumors (Smyth et al. 2020; Ferlay 2020. Accessed on September 30, 2021). In 2020, the global incidence of new cases exceeded one million (1,089,103), leading to 768,793 fatalities (Ferlay 2020. Accessed September 30, 2021). In recent years, the overall incidence and mortality of GC have shown a stable decrease, attributed to advancements in early diagnosis, standardization of treatment methods including surgery, neoadjuvant therapy, and targeted therapy (Yonemura et al. 2016; Tan 2019; Kawazoe et al. 2020; Sexton et al. 2020). Despite significant progress being achieved in the last decade, the long-term survival of patients with advanced GC still remains unsatisfactory. The 5-year survival rate following radical gastrectomy and chemotherapy for patients with GC varies from 30 to 50% (Cats et al. 2018; Al-Batran et al. 2019). Consequently, there is an urgent need to identify more precise and efficacious therapeutic targets.

The ubiquitin-conjugating enzyme E2 (E2) plays a crucial role in the ubiquitin–proteasome pathway by facilitating the transfer of ubiquitin to the substrate protein. As an E2 enzyme, UBE2L3 facilitates the ubiquitination process by catalyzing a variety of substrate proteins in conjunction with E1s and E3s. UBE2L3 is situated on chromosome 22 q11.2-13.1 and codes for 153 amino acid residues (Clague et al. 2015). An increasing body of research has demonstrated the involvement of UBE2L3 in the pathogenesis of various diseases through the regulation of protein stability. These diseases include systemic lupus erythematosus (SLE) (Kim et al. 2020; Mauro et al. 2023), hepatitis B virus (HBV) infection (Zhou et al. 2019), Alzheimer’s disease (AD) (Wei et al. 2019), ischemic stroke (IS) (Wei et al. 2019), celiac disease (CeD) (Fernandez-Jimenez and Bilbao 2019), and rheumatoid arthritis (RA) (Zeng et al. 2021). Furthermore, it has been observed that UBE2L3 exhibits abnormally high expression in various human tumor types, indicating a potential role for UBE2L3 as an oncogene. UBE2L3 has been demonstrated to facilitate the migration of cervical cancer cells (Weinberg et al. 2020; Yi et al. 2020a, b). Furthermore, several studies have suggested that the depletion of UBE2L3 inhibits the proliferation and induces apoptosis of hepatocellular carcinoma cells (Tao et al. 2020). Additionally, UBE2L3 in promoting the migration and invasion of lung cancer cells (Ma et al. 2022), and can be used as a potential response biomarker to enhance the efficacy of HSP90 inhibitors against lung adenocarcinoma (Marrugal et al. 2023). Another investigation demonstrated that UBE2L3 facilitated the cellular malignant characteristics of oral squamous cell carcinoma, such as proliferation, invasion, migration, and in vivo tumor growth (Cui et al. 2022). Nevertheless, the specific function of UBE2L3 in gastric cancer remains unclear.

This study aimed to examine the clinical significance of UBE2L3 expression in human GC tissues, explore the relationship between UBE2L3 expression and clinicopathological features, and assess its prognostic value using a multi-experimental approach.

## Materials and methods

### The analysis of gene expression data

The UBE2L3 gene expression analysis of various tumors can be accessed from the GEPIA database (http://gepia.cancer-pku.cn/index.html).

### Tissue specimens

Human tissue specimens were obtained from GC patients who underwent surgical excision at the Second Hospital of Lanzhou University between December 2016 and June 2017. The informed consent was signed by all patients, and the study protocol received approval from the Ethics Committee of the Second Hospital of Lanzhou University. The study included 125 patients with primary gastric cancer, with 11 patients excluded due to less than 5 years of follow-up. The cohort consisted of 114 patients, comprising 87 men and 27 women, all of whom did not undergo chemotherapy or radiotherapy before the surgical procedure, and all completed a 5-year follow-up period. All 114 specimens underwent confirmation through pathological examination and TNM staging.

### Cell culture

The six cell lines, namely GES-1, MKN45, MKN28, N87, AGS, and HGC27, were acquired from the Digestive Tumor Laboratory of Gansu Province. The six cell lines were cultured in RPMI 1640 medium (Gibco, USA) supplemented with 10% fetal bovine serum (FBS, Gibco, USA), and maintained in a humidified incubator at 37 °C with a 5% CO_2_ atmosphere.

### Lentiviral transfection

In the MKN45, MKN28, and AGS cell lines, the experimental group consisted of cells transfected with lentivirus obtained from GenePharma, China, while the negative control group comprised cells transfected with an empty lentiviral vector. Cell transfection was conducted using Invitrogen following the manufacturer’s protocol. Cells were cultured in six-well plates (Corning, USA) until reaching a cell density of 20–30%. Subsequently, the cells were harvested 18 h after transfection with lentivirus. After 72 h of continuous culture, the cells were subjected to puromycin screening for an additional 72 h. The transfection efficiency was assessed by observing green fluorescent protein expression under a microscope, and it was found that the fluorescence efficiency exceeded 80%, indicating a successful transfection.

### Cell viability assay

In this study, the cell proliferation potential of GC cells was assessed using the Cell Counting Kit-8 (CCK-8) assay kit (Solarbio, China). The cells (MKN45, MKN28, AGS) were seeded at a density of 1000 cells per well in 96-well plates (Corning, USA). The medium in each well was removed at different time intervals (0, 24, 48, 72, and 96 h). Subsequently, 10 μL of the prepared CCK-8 reagent was introduced into each well and incubated for 1 h at 37 °C in a light-sheltered incubator. Following incubation, the absorbance value (OD) was assessed at a wavelength of 450 nm.

### Colony-forming assay

The cells were cultured at a density of 500 cells per well in 6-well plates. The cells were cultured without disturbance for a period of two weeks to facilitate the formation of proliferating colonies. Subsequently, the proliferating cells underwent two gentle washes with phosphate-buffered saline (PBS) to eliminate any non-adherent cells. Subsequently, the cells were treated with paraformaldehyde for 15 min and then subjected to staining with 0.1% crystal violet (Solarbio, China) for an additional 15 min. The number of colonies was determined using Image J software.

### Cell invasion assay

The cells that were treated with trypsin were subsequently placed in the upper chamber of the transwell system (Corning, USA) that was coated with Matrigel (BD Bioscience). The cell count was 1 × 10^5^ cells per upper chamber. The cells underwent two gentle washes with PBS following 18–72 h of incubation. Subsequently, the cells underwent fixation and staining using the identical procedure employed in the colony formation assay. The number of invasive cells was visualized and quantified.

### Apoptosis assay

Apoptosis in GC cells was evaluated using flow cytometry. To analyze apoptosis, the apoptosis rate of adherent cells was assessed using the AnnexinV-APC/7-ADD apoptosis kit (Multisciences, China). The GC cell samples were suspended again in a binding buffer. 5 μl of Annexin V-APC and 10 μl of 7-AAD were added to each tube, followed by a 5-min incubation at room temperature in the dark. At the conclusion of the incubation period, the samples underwent processing using flow cytometry (Canto BD).

### Western blot analysis

Proteins were initially extracted using RIPA buffer containing a protease inhibitor cocktail (Beyotime), and the quantification of protein levels was determined using the BCA assay (Beyotime). Following the isolation of proteins, the entire protein content was separated using 10% SDS-PAGE and subsequently transferred to PVDF membranes for Western blot analysis. Subsequently, the membranes were blocked with 5% skim milk for 1 h, followed by sequential incubation with primary antibodies overnight and secondary antibodies for 1 h. To analyze and document the protein bands, the membrane underwent imaging using the enhancement chemiluminescence reagent (Biosharp, China) and a chemiluminescence imager (Tanon 4600, China). The antibodies used for Western blot analysis were as follows: anti-UBE2L3 (dilution 1:10,000, Abcam, USA) and anti-GAPDH (dilution 1:5000, ImmunoWay Biotechnology, USA).

### Quantitative real-time PCR (qRT-PCR)

The total RNA was isolated using AG RNAex Pro Reagent (Accurate Biology, China), followed by RNA conversion to cDNA, and subsequently subjected to quantitative real-time PCR using a CFX96 Touch qRT-PCR system (Bio-Rad). GAPDH was employed as an internal control for mRNA analysis. The values of the target genes were determined using the 2^−ΔΔCt^ method. The primer sequences have been documented in Table 1.

**Table 1:** Primer sequences in qRT-PCR

Primer	Sequences
UBE2L3-forward	5ʹ-GAAGCCAGCAACCAAAACCG-3ʹ
UBE2L3-reverse	5ʹ-TTCAGCTAGGTCAGCCCGAA-3ʹ
GAPDH-forward	5ʹ-GCACCGTCAAGGCTGAGAAC-3ʹ
GAPDH-reverse	5ʹ-TGGTGAAGACGCCAGTGGA-3ʹ

### Subcutaneous xenograft model in nude mice

In this investigation, two cohorts of stably transfected MKN45 cells (1 × 10^6^ cells) were subcutaneously administered to female Balb/c nude mice aged 5–6 weeks old, obtained from Chengdu Yaokang Biotechnology Co., LTD. Tumor dimensions were assessed at 3-day intervals over a period of 21 days. Subsequently, the mice were euthanized following inhalation of ether anesthesia, and the xenograft tumors were removed, weighed, and then stored in a refrigerator at − 80 ℃ after resection for future experiments.

### Immunohistochemical and scoring method

The immunohistochemical method was used to detect the expression level of UBE2L3 protein in human tissues and tumor tissues of nude mice. Tissue microarrays were created using gastric cancer (GC) tissues and adjacent normal tissues from 125 GC patients. Each sample was represented by two cores with a diameter of 1mm. Subsequently, the tumor tissue samples in nude mice were fixed, embedded in paraffin, and sectioned into 5 µm slices. Subsequently, the sections underwent antigen retrieval, followed by overnight incubation at 4 °C with anti-UBE2L3 antibody (1:500, Abcam, USA) and anti-Ki67 antibody (1:500, Servicebio, China). Following incubation with secondary antibodies, the sections underwent staining with 3,3ʹ-diaminobenzidine, and subsequent incubation with hematoxylin. Two pathologists assessed UBE2L3 immunostaining based on the percentage of stained cells and the intensity of staining in each section. The scoring system was determined by the proportion of stained cells in the positive area (0 = 0–5%, 1 = 6–25%, 2 = 26–50%, 3 = 51–75%, 4 = 76–100%) and the intensity of staining (0 = no staining, 1 = weak staining, 2 = moderate staining, 3 = strong staining). The immunohistochemical score is calculated by multiplying the score of the staining area by the score of the staining intensity. The samples were subsequently categorized as negative (-, 0 points), weakly positive (+ , 1–4 points), moderately positive (+ + , 5–8 points), and strongly positive (+ + +, 9–12 points).

### Statistical analysis

The study results were analyzed using GraphPad Prism (version 8.0, USA). The study utilized a Student’s t-test to compare two groups and a one-way analysis of variance (ANOVA) to compare multiple groups. The results of the study were reported as mean ± standard deviation (SD). *P* values less than 0.05 were deemed to be statistically significant. The chi-square test was employed to assess the correlation between UBE2L3 expression and clinicopathological data. Survival analysis was evaluated using the log-rank test.

## Results

### UBE2L3 gene expression in tumors

**Fig. 1:**
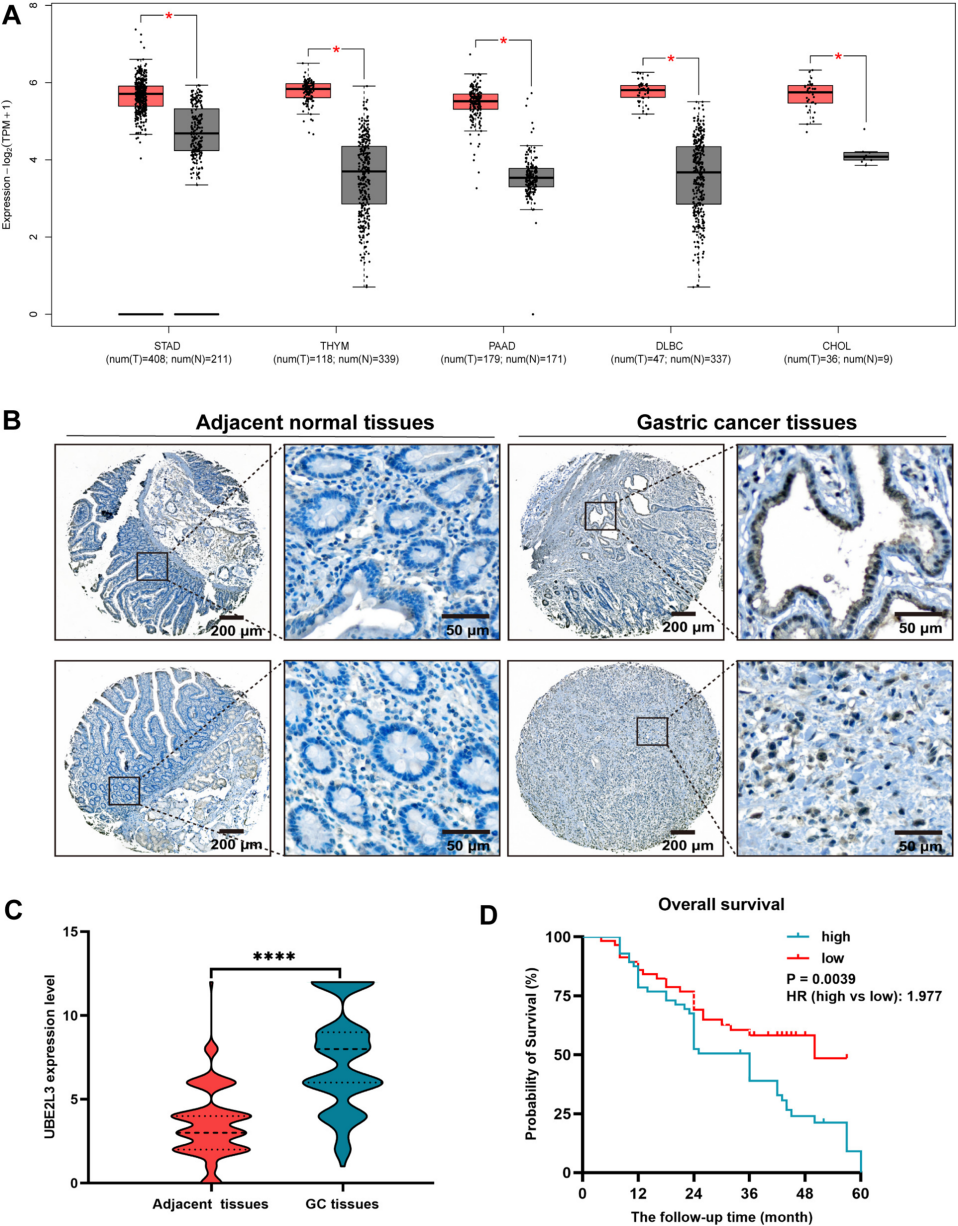
The expression of UBE2L3 in individuals diagnosed with gastric cancer and its association with prognosis. A UBE2L3 mRNA expression in different cancer tissues was assessed using the Gene Expression Profile Interaction Analysis (GEPIA) database. **B** Representative images illustrating UBE2L3 immunohistochemical staining in GC tissues and adjacent normal tissues. **C** The expression level of UBE2L3 in GC tissues was significantly higher compared to that in adjacent normal tissues. **D** High level of UBE2L3 was found to be correlated with a poorer overall survival (OS) in patients diagnosed with GC. The abbreviations STAD, THAM, PAAD, DLBC, and CHOL represent gastric adenocarcinoma, thymoma, pancreatic adenocarcinoma, diffuse large B-cell lymphoma, and cholangiocarcinoma, respectively

The GEPIA public database was utilized for the collection of UBE2L3 mRNA expression data in various tumor tissues, with the aim of further investigating the UBE2L3 mRNA expression in GC tissues. The various cancer tissues examined in this study comprised pancreatic cancer (*n* = 179) and normal pancreatic tissue (*n* = 171), gastric cancer (*n* = 408) and normal gastric tissue (*n* = 211), cholangiocarcinoma (*n* = 36) and normal bile duct tissue (*n* = 9), and normal pancreatic tissue (*n* = 10). Thymoma (*n* = 118) and normal thymic tissue (*n* = 339) were compared, as well as diffuse large B-cell tumor (*n* = 47) and normal tissue (*n* = 337). The data presented in this study provide insight into the high expression of UBE2L3 in various human cancers, suggesting that UBE2L3 may be involved in the pathogenesis of malignant tumors (Fig. 1A).

### Upregulation of UBE2L3 in gastric cancer tissues has been found to be associated with a poor prognosis for patients

**Fig. 2:**
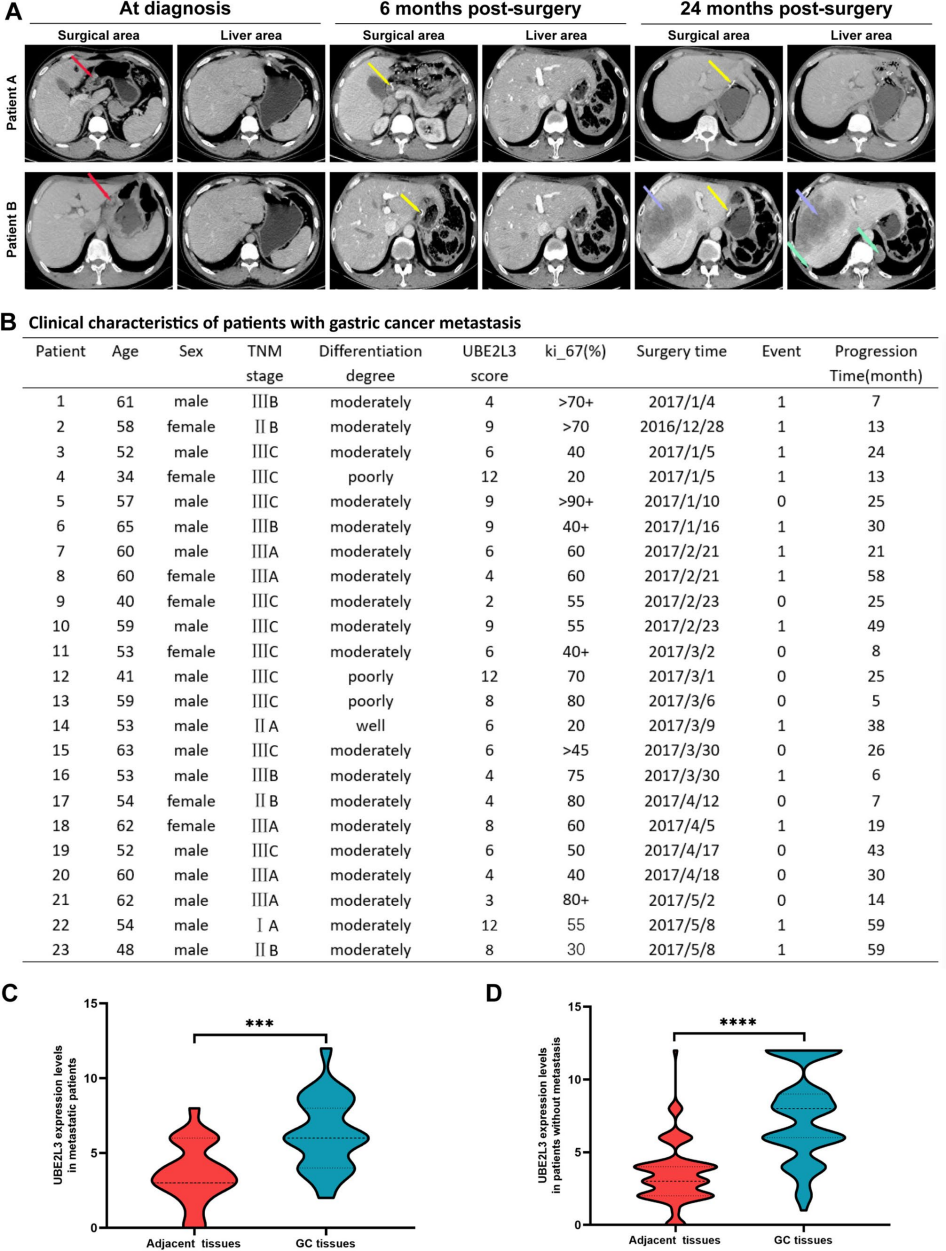
**A** The absence of GC metastasis in patient A during the follow-up period, whereas patient B developed liver and lung metastasis at 24 months (red arrow indicates the primary lesion, yellow arrow indicates the surgical site, blue arrow indicates liver metastasis, and green arrow indicates lung metastasis). **B** This section presents the clinical characteristics of 23 patients who experienced metastasis at various time points following their surgical procedures. **C** UBE2L3 expression levels of UBE2L3 in patients with metastasis. **D** UBE2L3 expression levels of UBE2L3 in patients without metastasis were examined

To clarify the potential clinical relevance of UBE2L3 in GC, we initially assessed the differential expression of UBE2L3 in GC tissues compared with adjacent normal tissues using immunohistochemical (IHC) staining on 125 specimens from 125 GC patients. As depicted in Fig. 1B, IHC data indicated that UBE2L3 is primarily expressed in the cytoplasm and nucleus of GC cells. The expression level of UBE2L3 in GC tissues was significantly higher than that in adjacent tissues (see Fig. 1C). Utilizing the median IHC score of UBE2L3, we segregated 114 GC patients into two categories: those with low UBE2L3 expression and those with high UBE2L3 expression. Table 2 illustrates the correlation between UBE2L3 expression and clinical pathological characteristics. The analysis of Table 2 reveals a strong correlation between the high expression of UBE2L3 in GC and the degree of differentiation (× 2 = 6.153, *P* = 0.0131) as well as TNM classification (× 2 = 6.216, *P* = 0.0447). However, no significant differences were observed in relation to age, gender, histological type, tumor size, and Lauren classification (refer to Table 2). As depicted in Fig. 1D, individuals exhibiting high UBE2L3 expression demonstrate a lower overall survival rate compared to those with low UBE2L3 expression. The CT data of the patients was collected over a 5-year follow-up period. In Fig. 2A, it was observed that Patient A did not develop metastases during the follow-up period, while Patient B was diagnosed with liver and lung metastases 24 months into the follow-up. A total of 23 patients were identified with metastases at various sites and at different times following surgery during the follow-up period. The specific patient data is depicted in Fig. 2B. Based on the IHC findings, the patients were categorized into two groups: those with metastasis and those without metastasis, and further divided into UBE2L3 high-expression group and UBE2L3 low-expression group. The findings indicated a significantly higher expression level of UBE2L3 in gastric cancer tissues compared to adjacent tissues in patients with and without metastasis, as illustrated in Fig. 2C and D. In summary, the heightened expression of UBE2L3 indicates an unfavorable prognosis in patients with GC.

**Table 2:** Relationship between UBE2L3 expression and clinicopathologic characteristics of patients with gastric cancer

Characteristics	UBE2L3 high expression (*n* = 57)	UBE2L3 low expression (*n* = 57)	Statistics	*P*
Age (years), mean ± SD	56.456 ± 9.849	56.719 ± 9.051	0.1485*	0.8822
Sex, *n* (%)				
Male	42 (74)	45 (79)	0.1941^†^	0.6595
Female	15 (26)	12 (21)		
Differentiation degree, *n* (%)				
Poorly differentiated	8 (14)	14 (25)	6.153^†^	0.0131
Moderately differentiated	37 (65)	40 (70)		
High differentiation	12 (21)	3 (5)		
Histological type, *n* (%)				
Intestinal	29 (51)	23 (41)	2.305^†^	0.3158
Diffuse	16 (28)	15 (26)		
Mixed	12 (21)	19 (33)		
Invasion depth (T grade), *n* (%)				
T1	10 (18)	8 (14)	3.143^†^	0.3701
T2	5 (9)	3 (5)		
T3	7 (12)	3 (5)		
T4	35 (61)	43 (76)		
Lymphatic metastasis (N grade), *n* (%)				
N0	22 (39)	13 (23)	3.340^†^	0.3421
N1	12 (21)	15 (26)		
N2	8 (14)	10 (18)		
N3	15 (26)	19 (33)		
TNM classification				
I	14 (25)	8 (14)	6.216^†^	0.0447
II	15 (26)	8 (14)		
III	28 (49)	41 (72)		
Tumor size				
≥ 5 cm	36 (63)	30 (53)	1.558^†^	0.2120
< 5 cm	21 (37)	27 (47)		

*t value. ^†^x^2^ values. SD: Standard deviation

### UBE2L3 expression in GC cells and construction of UBE2L3 knockdown and overexpress cells models

**Fig. 3:**
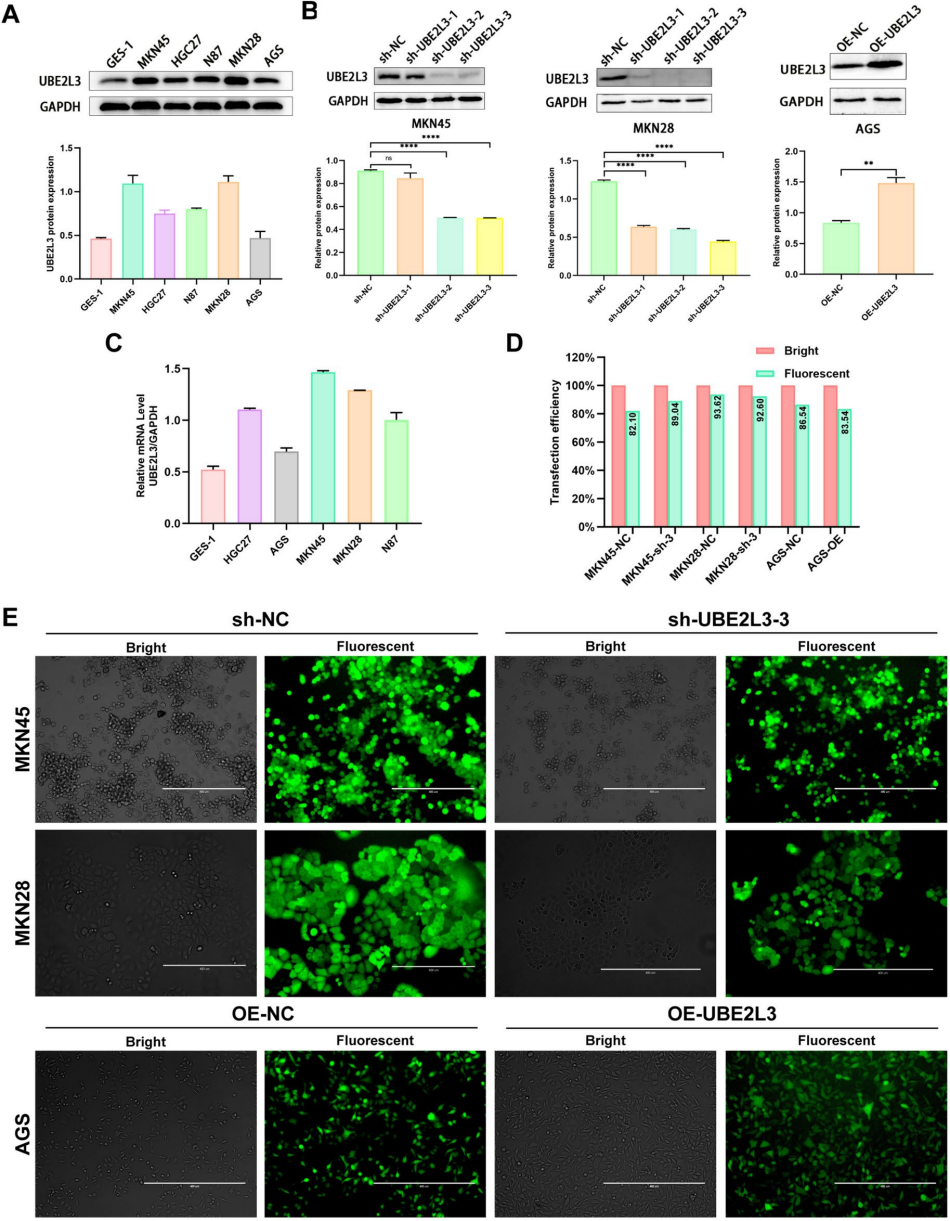
The expression of UBE2L3 in various cell lines and the establishment of a lentivirus transfection model. **A** The expression of UBE2L3 protein in six cell lines. **B** MKN45, MKN28, and AGS cells were transfected with lentivirus, and the expression of UBE2L3 was assessed using Western blot analysis. **C** UBE2L3 mRNA expression of UBE2L3 was assessed in six different cell lines. **D** Transfection efficiency of lentivirus: the transfection efficiency of lentivirus in three cell lines was found to be above 80%. **E** 72 h after transfection, the fluorescence of the green fluorescent protein on the lentiviral vector was observed

The expression of UBE2L3 in GC was assessed by conducting Western blot analysis and quantitative real-time polymerase chain reaction (qRT-PCR) on five human GC cell lines and GES-1. The results indicate that UBE2L3 exhibited high expression in MKN45 and MKN28, and low expression in AGS (Fig. 3A, C). Subsequently, three UBE2L3 shRNAs were employed to suppress the expression of UBE2L3 in MKN28 and MKN45, while UBE2L3 was overexpressed in AGS to ascertain its function in GC. The transfection efficiency was assessed by observing the presence of green fluorescent proteins in MKN28, MKN45, and AGS cells. The experimental results indicated that the transfection efficiency exceeded 80% in all three cell lines (Fig. 3D, E). Furthermore, the transfection efficiency was assessed through Western blot analysis, revealing that two UBE2L3 knockdown cell lines and one UBE2L3 overexpressing cell line successfully attained the anticipated UBE2L3 protein levels (Fig. 3B). The aforementioned data suggests that the establishment of UBE2L3 knockdown and overexpression cell models has been successful, rendering them suitable for subsequent experiments.

### UBE2L3 regulates GC cells proliferation, colony formation, invasion, and apoptosis

**Fig. 4:**
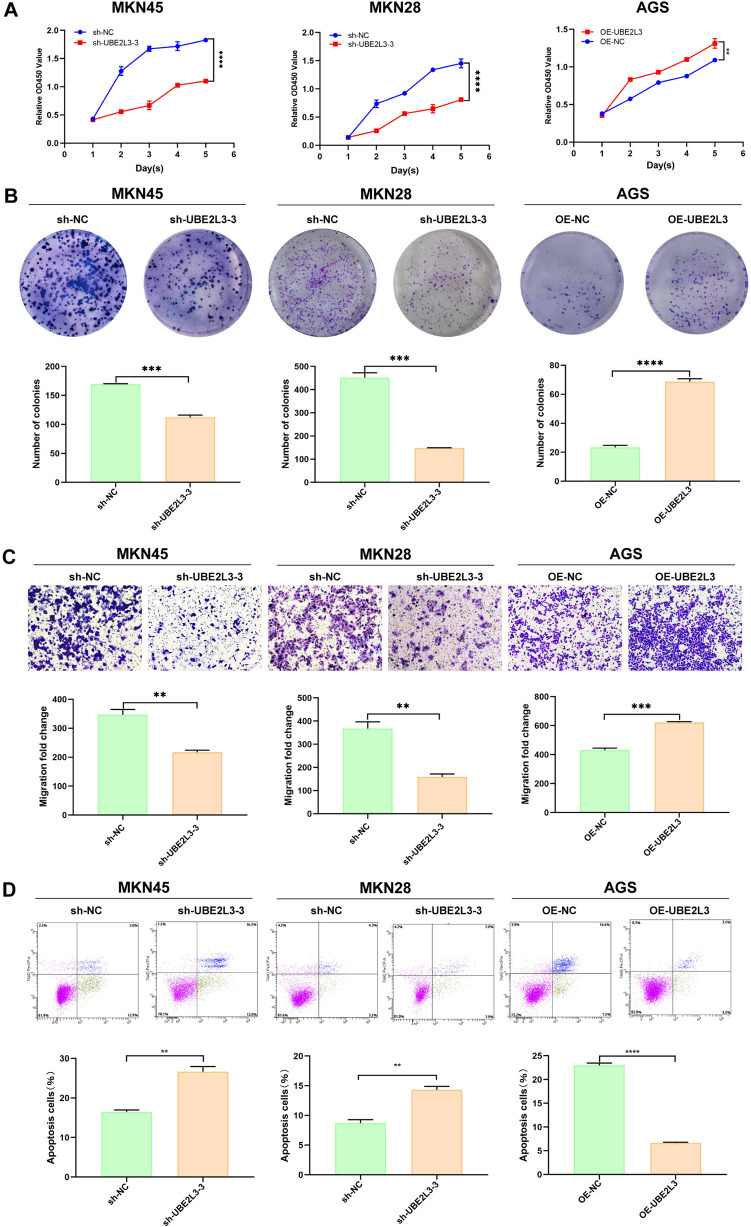
The impact of UBE2L3 on the functionality of GC cell lines. **A** Cell Counting Kit-8 (CCK-8) assay. **B** The colony formation assay was conducted. **C** Transwell assay was conducted to assess invasion. **D** Cell apoptosis assay

Based on the discovery that UBE2L3 levels are linked to the prognosis of GC, we employed the following experimental approach to validate the functional role of UBE2L3 in the malignant behavior of GC in vitro. The study revealed that UBE2L3 promotes the proliferation rate of GC cells, as demonstrated by the CCK-8 assay results presented in Fig. 4A. Furthermore, the cells’ proliferation ability was verified through the colony formation assay. A reduced number of colonies were observed in the UBE2L3 knockdown group, while the overexpression group exhibited the opposite outcome (Fig. 4B). Subsequently, the Transwell assay was used to evaluate the invasive capacity of GC cells. Likewise, the suppression of UBE2L3 hindered cellular passage through the matrigel, whereas the up-regulation of UBE2L3 facilitated increased cellular invasion through the matrigel (Fig. 4C). Finally, the results of the apoptosis assay revealed a significant increase in the apoptosis rate of the sh-UBE2L3 group, and a significant decrease in the UBE2L3 overexpression group (Fig. 4D). In conclusion, the aforementioned findings unequivocally demonstrate the significant involvement of UBE2L3 in GC.

### UBE2L3 promotes tumor growth in vivo

**Fig. 5:**
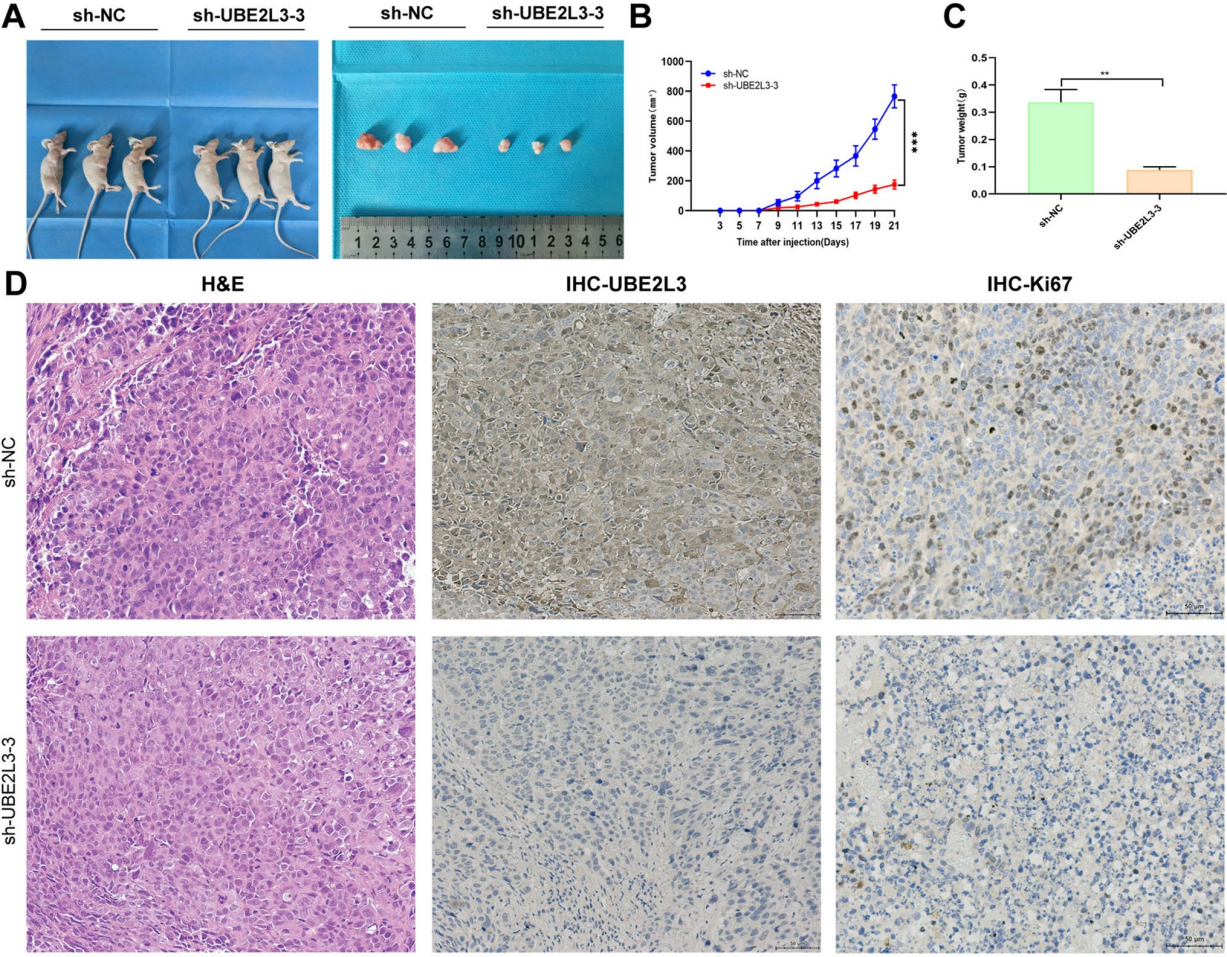
UBE2L3 enhances the growth of GC in an in vivo setting. **A** Inhibition of UBE2L3 knockdown suppressed the growth of GC in vivo. **B** Graphs depicting the growth of tumors over time. **C** Weight of the tumor. **D** Immunohistochemistry (IHC) staining was performed to detect the expression of UBE2L3 and Ki67 in xenograft tumors

To validate the findings of the in vitro experiments, cell-derived xenograft (CDX) models were established in nude mice through the injection of MKN45 sh-NC and MKN45 sh-UBE2L3-3 cells. The growth of subcutaneous tumors in nude mice was monitored every 3 days, and the changes in tumor volume were measured and recorded (Fig. 5B). The results demonstrate that the tumor volume of the sh-NC group was greater than that of the sh-UBE2L3-3 group (Fig. 5A). Following the euthanasia of the nude mice, the tumor tissues were extracted, weighed, and the dimensions of each tumor were recorded. The findings indicated that the tumors in the sh-UBE2L3-3 group exhibited reduced size and weight compared to those in the sh-NC group (Fig. 5C). Finally, the findings from the hematoxylin and eosin (H&E) staining and immunohistochemistry (IHC) analysis (Fig. 5D), conducted on tumor tissues, revealed that the levels of UBE2L3 and Ki67 expression were higher in the sh-NC group compared to the sh-UBE2L3-3 group. The collective data presented above suggest that the suppression of UBE2L3 hinders the growth of gastric cancer in an in vivo setting.

## Conclusion

The human genome contains an estimated 40 genes that encode E2s. The genes were categorized into four classes and exhibited a conserved domain that included catalytic Cysteine residues (Stewart et al. 2016; Welsh et al. 2022). In recent years, there has been increasing research interest in the role of E2s in tumors. UBE2T, functioning as an E2 enzyme, has been identified as a crucial regulator of tumor advancement in various types of cancer, including gastric cancer (Yu et al. 2021), hepatocellular carcinoma (Sun et al. 2020), pancreatic cancer (Jiang et al. 2023), lung adenocarcinoma (Zhu et al. 2021), retinoblastoma (Xu et al. 2022), and glioblastoma (Huang et al. 2020). Furthermore, Qi et al. reported that the overexpression of UbcH5c was linked to a negative prognosis in pancreatic cancer (Qi et al. 2022). Another study has confirmed that UBE2J1 inhibits the progression of colorectal cancer by enhancing the ubiquitination and degradation of RPS3 (Wang et al. 2023). Furthermore, UBE2M and UBE2F, functioning as E2 enzymes, facilitate the transfer of NEDD8 to NEDD8 E3 ligase (Zheng et al. 2021). Several studies have demonstrated their overexpression in various types of cancer, such as hepatocellular carcinoma, breast cancer, lung adenocarcinoma, osteosarcoma, and esophageal squamous cell carcinoma (Zhou et al. 2017; Heo et al. 2020; Wang et al. 2020; Zheng et al. 2021). Moreover, an additional E2 enzyme, UBE2C, has been found to facilitate the proliferation and viability of lung carcinoma cells harboring Kras mutations. UBE2C was also found to be essential for the development of Krasg12d-induced lung tumors (Zhang et al. 2023). Regarding UBE2L3, its biological functions have been reported in hepatocellular carcinoma, cervical cancer, and lung adenocarcinoma (Tao et al. 2020; Yi et al. 2020a, b; Marrugal et al. 2023).

The study revealed that the expression levels of UBE2L3 mRNA were significantly elevated in the majority of gastric cancer tissues compared to adjacent non-tumor gastric tissues, as determined through analysis of publicly available databases. Furthermore, we verified the increased expression of UBE2L3 in 125 GC tissues compared to adjacent tissue through immunohistochemistry analysis. Significantly, it was observed that elevated UBE2L3 expression was associated with the degree of differentiation, TNM classification, and prognosis in patients with GC. Furthermore, out of the 125 patients, 23 had metastasis. The expression level of UBE2L3 in GC tissues was significantly higher than that in adjacent normal tissues, both in patients with and without metastasis. Subsequently, the biological function of UBE2L3 in gastric cancer was validated through a series of laboratory methods. The findings indicated that UBE2L3 facilitated the proliferation, clone formation, and invasion of GC cell lines, while suppressing the apoptosis of GC cell lines. In vivo experiments, UBE2L3 was found to promote tumor progression. The aforementioned findings indicate that UBE2L3 functions as an oncogene in GC.

To date, research on the molecular mechanisms of UBE2L3 in diseases has primarily concentrated on the following areas. First, UBE2L3 is involved in the regulation of the NF-κB signaling pathway. It has been reported that UBE2L3 significantly enhances NF-κB activation induced by TLR7 in SLE patients through its interaction with LUBAC (Mauro et al. 2023). Taehyeung Kim and colleagues observed an up-regulation of UBE2L3 and a down-regulation of TNFAIP3 in the context of NF-κB activation, which subsequently led to the induction of inflammatory factors, suggesting a synergistic role (Kim et al. 2020). Second, the process of p53 polyubiquitination mediated by UBE2L3 has been found to decrease p53 stability during the progression of cervical cancer (Yi et al. 2020a, b). Third, UBE2L3 was involved in the process of lysophagy and the ubiquitination of lysosomes in conjunction with UBE2N (Shima et al. 2023). Finally, the suppression of UBE2L3 resulted in an elevation of non-homologous end joining and a reduction in homologous recombination during double-strand break repair, achieved through the regulation of 53BP1 protein levels (Han et al. 2014; Pozo et al. 2017). Nevertheless, further research is required to elucidate the mechanism through which UBE2L3 facilitates tumor initiation and progression in gastric cancer.

Recent clinical applications have confirmed the effectiveness of HER2 in targeted therapy for GC; however, the potential audience for this treatment remains limited. In the future, the treatment of gastric cancer is expected to involve a combination of targeted therapies and a gradual shift toward personalized treatment approaches. The identification of UBE2L3 and the prospect of further comprehensive investigation suggest the potential for significant advancements in targeted therapy for gastric cancer.

### Supplementary Information

Below is the link to the electronic supplementary material.

Supplementary file1 (RAR 23458 KB)

## Data Availability

The data generated and analyzed in this study were available.
